# A Prospective Randomized Controlled Trial of AJG522 versus Standard PEG + E as Bowel Preparation for Colonoscopy

**DOI:** 10.1155/2015/521756

**Published:** 2015-01-22

**Authors:** Toshihiko Sagawa, Ken Sato, Taku Tomizawa, Masafumi Mizuide, Hidetoshi Yasuoka, Yasuyuki Shimoyama, Shiko Kuribayashi, Satoru Kakizaki, Osamu Kawamura, Motoyasu Kusano, Masanobu Yamada

**Affiliations:** ^1^Department of Medicine and Molecular Science, Gunma University Graduate School of Medicine, 3-39-22 Showa-machi, Maebashi, Gunma 371-8511, Japan; ^2^Department of Endoscopy and Endoscopic Surgery, Gunma University Hospital, 3-39-15 Showa-machi, Maebashi, Gunma 371-8511, Japan

## Abstract

Polyethylene glycol- (PEG-) based bowel preparations for colonoscopies are often poorly tolerated due to the large volumes of fluid intake required. We compared low-volume “modified” PEG + ascorbic acid (AJG522) with standard PEG with electrolytes (PEG + E) in addition to a stimulant laxative and an agent to improve bowel function for the bowel cleansing before colonoscopy to evaluate its efficacy, safety, and acceptability. Outpatients scheduled to undergo colonoscopy were randomized to receive either AJG522 or PEG + E. Bowel cleansing conditions were assessed via macroscopic fecal findings by blinded and independent investigators. A survey of the patients' feedback regarding the preparation was conducted by questionnaire. Successful cleansing was achieved in all cases, except for 4 cases in the PEG + E group, at 3 hours after taking the preparation. The fecal properties were significantly clearer in the AJG522 group than in the PEG + E group at 2 hours after taking each preparation (*P* = 0.013). Although the total liquid volume of the bowel preparation was not reduced, the AJG522 preparation could significantly reduce the required volume of the preparation (*P* < 0.0001). Moreover, the patients in the AJG522 group had better acceptability (*P* = 0.010). There were no significant differences in the safety profiles between groups (UMIN000013892).

## 1. Introduction 

The widely used polyethylene glycol- (PEG-) based preparations have been shown to be highly effective for bowel cleansing when taken as instructed and under ideal conditions [1, 2]. Standard PEG with electrolyte (PEG + E) preparations typically requires the consumption of approximately 4 L of fluid for bowel preparation. However, many people perceive this volume of the solution to be unpleasant or intolerable. Recently, the addition of ascorbic acid to PEG (PEG + Asc, Moviprep, Norgine, Harefield, UK) has been found to reduce the effective volume of the bowel cleansing solution by up to 2 L [3, 4]. However, until recently, PEG + Asc was not available in Japan. In Japan, the combination of an agent to improve bowel function [5] and/or a stimulant laxative [6] has allowed the volume of standard PEG + E preparations (PEG + E, Niflec, Ajinomoto Co., Inc., Tokyo, Japan) to be reduced to approximately 2 L [5].

Beginning in June 2013, AJG522 (Moviprep, Ajinomoto Co., Inc., Tokyo, Japan), which is essentially similar to the PEG+Asc solution (Moviprep, Norgine) that is available in the EU and North America, was placed on the market in Japan as a “modified” PEG + Asc. In AJG522, Macrogol (PEG 3350), one of the main active ingredients of PEG + Asc, was replaced by Macrogol 4000, in accordance with the Japanese Pharmacopoeia. Currently, one single-arm trial [7] regarding the safety and efficacy of AJG522 as a bowel cleansing agent for colonoscopy has been reported. Moreover, a comparable study between the AJG522 and PEG + E preparations was performed in rats [8]. However, no comparable clinical studies have been undertaken to make more meaningful comparisons.

Our study was designed to evaluate the efficacy, safety, and acceptability of the AJG522 preparation compared to the standard PEG + E preparation. To encourage defecation, an agent to improve bowel function and a stimulant laxative were used in combination with each bowel preparation.

## 2. Patients and Methods

### 2.1. Ethical Validity

Our study was conducted in accordance with the Declaration of Helsinki and was approved by the institutional review board of Gunma University. Written informed consent was obtained from all participating subjects.

### 2.2. Inclusion and Exclusion Criteria

Adult Japanese outpatients 20 years of age or older requiring a total colonoscopy were recruited from our university hospital from July 2013 to December 2013. All of the patients had at least one prior colonoscopy experience. This was required for the following reasons: (1) acceptability and tolerability for not only the bowel preparation but also the colonoscopy itself may vary widely among individuals if the subjects are colonoscopy-naïve patients; and (2) there may be significant differences in acceptability and tolerability between colonoscopy-naïve patients and colonoscopy-experienced patients. We included consenting study subjects unless they had any of the following: a history of colonic resection; ileus; gastrointestinal obstruction or perforation; severe inflammatory bowel disease such as toxic megacolon; heart failure (New York Heart Association [NYHA] Class III or IV); acute life-threatening cardiovascular disease; untreated or uncontrolled hypertension; severe liver failure; severe psychosomatic disease; or end-stage renal insufficiency. All hospitalized patients were excluded because they have poor statuses in general. For example, some patients are given laxatives through a nasogastric tube, suffer severe diarrhea, or experience long fasting durations. Poor general status often prevents the completion of a colonoscopy. Thus, patient backgrounds can be quite different between outpatients and hospitalized patients. Women were excluded from the study if they were pregnant, breastfeeding, or at risk of becoming pregnant. Moreover, patients who might have been at risk if they entered the trial were also excluded based on the opinion of the investigator.

### 2.3. Treatment Allocation and Masking

Eligible study subjects were randomly assigned to one of two preparations [AJG522 or standard PEG + E]. The study subjects were allocated using a computer-generated, randomized code list with a block size of 4. Eligible patients were assigned to the next available randomization number. The assigned preparation was dispensed by a coresearcher who was not involved with the colonoscopy. Neither of the researchers who performed the colonoscopy nor the estimator of the fecal properties knew the preparation allocation.

### 2.4. Bowel Cleansing Plans and Dietary Limitations

The study preparation given was either AJG522 (containing 100.0 g macrogol 4000, 7.5 g sodium sulfate, 2.7 g sodium chloride, 1.0 g potassium chloride, 4.7 g ascorbic acid, 5.9 g sodium ascorbate, and green apple flavoring per liter; total dose = 2 L) or PEG + E (containing 59.0 g macrogol 4000, 5.7 g sodium sulfate, 1.5 g sodium chloride, 0.7 g potassium chloride, 1.7 g sodium bicarbonate, and lemon flavoring per liter; total dose = 2 L). The study subjects were scheduled for colonoscopies in the afternoon. They were instructed to take 3 tablets (15 mg) of an agent to improve bowel function, mosapride citrate (Gasmotin, Sumitomo Dainippon Pharma Co., Ltd., Osaka, Japan) and 2 tablets (24 mg) of a stimulant laxative, sennosides A and B (Pursennid, Novartis Pharma K.K., Tokyo, Japan) in the evening before the day of their colonoscopy. Each patient also took 3 tablets (15 mg) of mosapride citrate in the morning on the day of their colonoscopy. Essentially, the instructions for the corresponding preparations complied with those of the manufacturer. Specifically, as opposed to PEG + E, which is an isotonic solution, AJG522 is a hypertonic solution. Therefore, an amount of water equal to half the volume of the AJG522 preparation is recommended to be taken, according to the manufacturer's instructions, to avoid dehydration; that is, in the AJG522 group, each patient took AJG522 at a rate of 1000 mL per 60 min and subsequently ingested water at a rate of 500 mL per 30 min. Then, each patient took AJG522 at a rate of 500 mL per 30 min and, subsequently, water at a rate of 250 mL per 15 min. Next, each patient repeated this cycle, alternating between AJG522 and water as done previously. In the PEG + E group, the corresponding preparations were taken at a rate of 250 mL per 15 min during the morning on the day of their colonoscopy. Importantly, the rate of ingesting each liquid was fixed to 250 mL per 15 min in both groups. Each patient stopped drinking when the bowel cleansing conditions reached grade A or B, as mentioned below, or at 3 hours after taking the bowel preparation. Importantly, in the AJG522 group, the patients who stopped drinking when the bowel cleansing conditions reached grade A or B did not always drink an amount of water equal to half of the volume of the AJG522 preparation. The bowel cleansing plans for each group are shown in Figure 2. The bowel cleansing efficacy was assessed based on the macroscopic fecal findings at 2 and 3 hours after taking each preparation. The preparation volume and the total liquid volume were also evaluated.

There were no stringent dietary limitations on the day before the colonoscopy, although all patients received nutritional guidance to eat a low-residue diet. Thus, a diet for the examination was not mandatory for the patients. All patients did adhere to a diet for the examination or a self-selected individual diet before 21:00 on the day before the colonoscopy. From that point on, they were food-deprived until after the colonoscopy. The colonoscopy was performed at any time after the bowel cleansing conditions reached grade A or B based on the predefined scoring system (Figure 3) or when the conditions were evaluated in the afternoon, 3 hours after taking the preparation.

### 2.5. Assessments

The primary endpoint was the fecal properties of each colonoscopy bowel cleansing condition via macroscopic stool observation. Assessment of the macroscopic fecal findings was performed by blinded and independent researchers who did not recruit the patients for the study, were not involved in the allocation, did not know the assignment of the preparations or the reason for the colonoscopy, and did not perform the endoscopy. Each fecal finding was described based on a predefined scoring system (Figure 3). The total volume of bowel preparation ingested by the study subjects was evaluated by the coresearchers. The coresearchers, who did not know the preparation assignment or the reasons for the colonoscopy, evaluated the bowel cleansing conditions during the colonoscopy.

The secondary endpoints included taste, acceptability, willingness to repeat, patient satisfaction, and adverse events, based on a questionnaire of the bowel preparation experiences of each patient. The ingestion of food the day before the colonoscopy, which could affect the bowel cleansing efficiency, was also evaluated by the questionnaire. The researchers who performed the endoscopy had no knowledge of this information or of the assigned preparations before the procedure, although they did know the reason for the colonoscopy. The full questionnaire is shown in the following.

*Patient Questionnaire*.

(Q1). Did you eat the diet of examination the day before the colonoscopy (please tick)?
   □ Yes
   □ No
(Q2). How did the preparation taste (please tick)?
   □ Good
   □ Satisfactory
   □ Neutral
   □ Unsatisfactory
   □ Bad
(Q3). Could you take more preparation after your stool become clear liquids or at 3 hours after taking the preparation if not (please tick)?
    □ Yes
   □ No
(Q4). Did you have willingness to repeat the same preparation for the next colonoscopy (please tick)?
    □ Yes
   □ Acceptable
   □ Neutral
   □ No
(Q5). Did you satisfy the bowel preparation in view of time (please tick)?
   □ Satisfactory
   □ Neutral
   □ Unsatisfactory
(Q6). Tick any adverse events experienced during the preparation period:
   □ Vomiting
   □ Nausea
   □ Bloating
   □ Cramps
   □ Dizziness
   □ Insomnia
   □ Pain
   □ Anal Irritation
   □ Thirst
   □ Breathlessness
   □ Other symptom
   (Please specify, if any…………………).

### 2.6. Sample Size and Statistical Analysis

No formal sample size calculations were made for the evaluation of the primary endpoint, as this was a preliminary study to assess the relative performance of the two preparations. However, rough sample size estimations were attempted. In our preliminary trial, the rate of the patients with fecal properties that reached grade A at 2 hours after drinking the preparation were 8 out of 10 and 4 out of 10 subjects in the AJG522 and PEG + E groups, respectively. These estimations were based on the ability to detect a difference in the bowel cleansing efficiency of 40% (e.g., 80% versus 40%), with 80% power and a 2-sided *α*-level of 0.05. This resulted in a total sample size of 56 patients (28 patients per preparation group). However, this estimation was based on a very small sample size based on the preliminary trial and may lack accuracy. We needed to recruit at least 60 evaluable patients (at least 30 patients per preparation group). To ensure 60 evaluable patients, even with the consideration of an unexpectedly large number of dropouts, we recruited 80 patients.

Continuous data are presented as the medians and ranges. The difference in the distribution of the bowel cleansing grading between the preparation groups was analyzed using a Wilcoxon signed rank test. For each of the secondary efficacy variables from the patient questionnaire (taste, acceptability, willingness to repeat, patient satisfaction, and adverse events), the difference in the distribution of the categories between the preparation groups was analyzed using a Wilcoxon signed rank test. Differences in the proportions were measured by Fisher's exact test. Continuous variables were compared using the Mann-Whitney *U* test. The results were considered statistically significant when the *P* value was <0.05.

## 3. Results

### 3.1. Patient Characteristics

**Figure 1:**
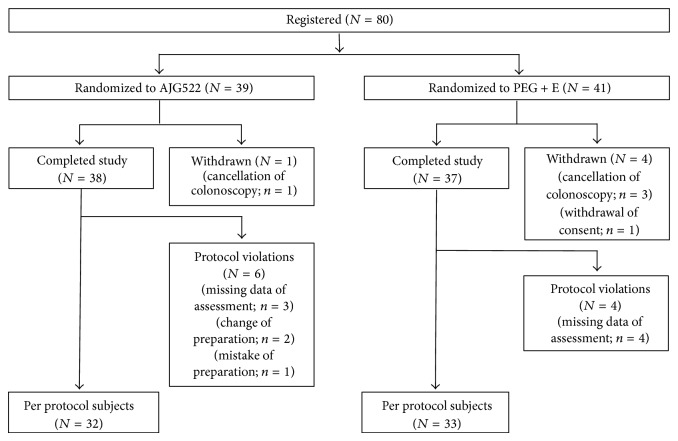
Patient flow during the study (CONSORT diagram).

**Figure 2:**
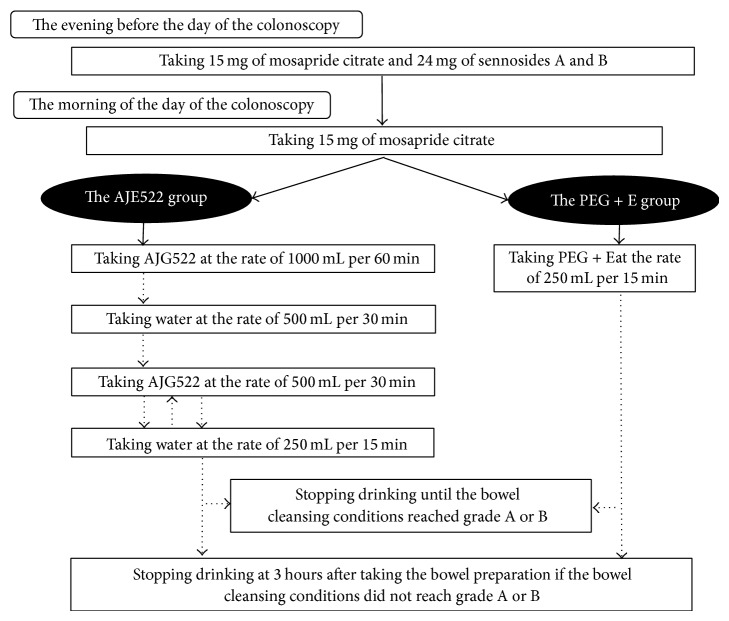
Bowel-cleansing plans for each group.

**Figure 3:**
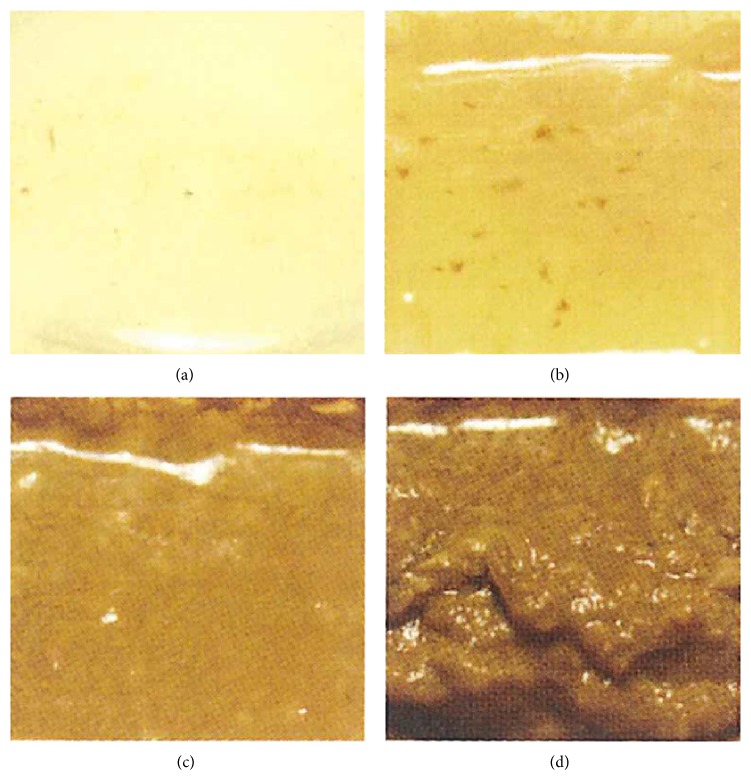
Gradingscore of the fecal properties (a–d). Fecal properties representative of each cleansing condition. (a) Clear liquid. (b) Brown liquid with insoluble residue. (c) Semisolid, only partially removable muddy stools. (d) Irremovable, hard, and heavy stools.

A total of 80 study subjects (39 AJG522, 41 PEG + E) were registered and randomized, 75 of whom completed the study preparations (38 AJG522, 37 PEG + E). The remaining subjects were withdrawn due to a cancellation of their colonoscopy or withdrawal of their consent. Thus, 75 subjects comprised the intention-to-treat population. Among these, 6 subjects had protocol violations due to missing assessment data, preparation changes, or preparation mistakes in the AJG522 group, whereas 4 had missing assessment data in the PEG + E group. The per protocol population used for the analysis comprised 65 patients (32 AJG522, 33 PEG + E; see Figure 1 [CONSORT diagram]). There were no significant differences between the preparation groups at baseline in terms of demographics, the reasons for the colonoscopy, or the comorbidities (Table 1). 

### 3.2. Ingestion of a Diet for the Examination

Patients were asked whether they ate a diet for the examination the day before the colonoscopy using a 2-point verbal scale of “Yes” or “No.” In the AJG522 group, 18.0% patients answered “Yes,” whereas, in the PEG + E group, 3% answered “Yes.” However, there was no significant difference (*P* = 0.054) in the proportions of patients consuming a diet for the examination between the preparation groups.

### 3.3. Bowel Cleansing Efficacy

The fecal properties at 2 hours after drinking the preparation were significantly better in the AJG522 group than in the PEG + E group (Table 2). There was a trend towards better fecal properties in the AJG522 group at 3 hours after taking each preparation (Table 2,  *P* = 0.062). The median volumes of the preparations consumed by the patients whose fecal properties reached grade A or B or who took the preparations for 3 hours without reaching grade A or B were 1000 (range 1000–2000) mL and 2000 (range 1150–2000) mL in the AJG522 and PEG + E groups, respectively (*P* < 0.0001) (Table 2). However, because patients drank extra water, as recommended by the manufacturer's documents to avoid dehydration, as mentioned previously, the total median liquid volume consumed by the patients whose fecal properties reached grade A or B or who took the preparations for 3 hours without reaching grade A or B was 1500 (range 1250–3000) mL in the AJG522 group. This value did not differ significantly from the total volume of preparations ingested in the PEG + E group (*P* = 0.088) (Table 2).

All cases in which the fecal properties reached grade A or B could be rated as the level at which a colonoscopy could be performed without any problem. An enema was administered to 4 patients whose fecal properties remained at grade C at 3 hours after drinking the PEG + E preparation.

### 3.4. Patients' Feedback

A summary of the patients' feedback is shown in Table 3. Patients rated the taste of the bowel preparation using a verbal scale of “Good,” “Satisfactory,” “Neutral,” “Unsatisfactory,” or “Bad.” In the AJG522 group, 28.1% of the patients rated the taste as good, compared with 9.1% in the PEG + E group, whereas the proportions of patients rating the taste as bad were 3.1% and 6.1%, respectively. As a whole, there was no significant difference in the proportions of the patients' taste ratings between the preparation groups.

Acceptability was evaluated by the willingness of the subjects to consume more of the preparation after the bowel cleansing conditions had reached grade A or B or at 3 hours after taking the preparation if they had not reached grade A or B. Patients were asked if they could ingest more preparation after their stool became a clear liquid using a 2-point verbal scale of “Yes” or “No.” In the AJG522 group, 65.6% patients answered “Yes,” whereas in the PEG + E group, only 33.3% answered “Yes” (*P* = 0.010). Thus, the acceptability was significantly better in the AJG522 group than in the PEG + E group.

Patients were asked to assess their willingness to repeat the same preparations for the next colonoscopy using a 4-point verbal scale of “Yes,” “Acceptable,” “Neutral,” or “No.” There was no significant difference in the proportions of patients who were willing to repeat the same preparations between the preparation groups.

Patient satisfaction was evaluated by their satisfaction with the bowel preparation regarding the time. Thus, patients were asked to describe their satisfaction using a 3-point verbal scale of “Satisfactory,” “Neutral,” or “Unsatisfactory.” All patients in whom the fecal properties did not reach grade A at 3 hours after taking the preparation answered “Unsatisfactory.” In the AJG522 group, 46.9% patients answered “Satisfactory,” compared with 24.2% in the PEG + E group, whereas the proportions of patients who answered “Unsatisfactory” were 25.0% and 30.3%, respectively.

### 3.5. Adverse Events

Both of the preparations were generally well tolerated. No serious adverse events were observed. Via the questionnaire, 3 adverse events (vomiting, nausea, or thirst) were reported in 5 patients in the AJG522 group, and 2 adverse events (vomiting or thirst) were observed in 7 patients in the PEG + E group. The other adverse events such as abdominal pain or eruptions were not observed. All adverse events were rated as mild. The numbers of cases of vomiting or nausea were similar in both preparation groups (vomiting: AJG522 1, PEG + E 0; nausea: 2 and 5, resp.), as were the numbers of cases of thirst (2 and 2, resp.). There was no significant difference in the safety profiles between the groups.

## 4. Discussion

Our main result is that the bowel cleansing efficacy was significantly superior in the AJG522 group compared with the standard PEG + E group regarding time. This conclusion was based on the results regarding the bowel cleansing conditions at 2 hours after consuming the preparation. Moreover, the AJG522 preparations had better acceptability, as evaluated by the willingness of this group to consume more of the preparation after the bowel cleansing conditions had reached grade A or B or at 3 hours after taking the preparation if they had not reached grade A or B. PEG + Asc, originally developed by Norgine, has been marketed in Europe and North America and is the most frequently used bowel preparation for colonoscopy worldwide. Based on PEG + Asc, AJG522 was developed to appeal to the Japanese population by altering the macrogol content, flavoring substances, and agents. Thus, the composition of AJG522 is different from that of the PEG + Asc that is available in Europe and North America. This is the first prospective study to compare AJG522 and standard PEG + E and, thus, deserves particular consideration.

There was a trend towards increasing the rate of taking a diet for the examination in the AJG522 group than the standard PEG + E group, although there was no significant difference between the groups. However, all patients received nutritional guidance to eat a low-residue diet on the day before the colonoscopy. Thus, we think that the role of a diet for the examination on efficiency in bowel cleansing was limited.

In a rat model, the volume of fecal water in the intestinal contents was shown to be significantly increased in weight in the AJG522 versus standard PEG + E-treated groups. AJG522 significantly decreased the number of doses and shortened the elapsed time after the first administration until the initiation of the excretion of watery diarrhea compared with PEG + E [8]. These results are consistent with our data. A single-arm trial [7] assessing the safety and efficacy of AJG522 as a bowel cleansing agent for colonoscopy showed that successful intestinal cleansing was achieved in 100% participants and that the preparation was well tolerated, except for one incomplete participant with a mild eruption that naturally resolved without treatment. Although no subject experienced an eruption in our study, the sufficient bowel cleansing efficacy and safety were consistent with our data.

Much effort has been made to decrease the total liquid volume of such preparations in various previous studies. One of the methods used suggests using 2 L of PEG + Asc instead of 4 L of PEG + E [6, 9]. Previous studies have shown that the low-volume of PEG + Asc was better tolerated and had a superior bowel cleansing capacity than the large volume of PEG + E. Another method calls for the use of the stimulant laxative sennosides or the digestive tract functional improvement agent mosapride citrate in addition to low-volume PEG [4, 5, 10]. However, one study [10] showed that a low-volume PEG plus sennosides preparation (120 mg oral sennosides syrup followed by 2 L PEG + E) was better tolerated but had an inferior efficacy compared with the standard large-volume PEG + E preparation (4 L PEG). By contrast, Tajika et al. reported that a mosapride citrate plus PEG + E preparation had a superior bowel cleansing efficacy compared with a placebo plus PEG + E preparation regarding the quality and time required [5]. Another method involved the use of combined oral sennosides with magnesium (Citramag) and at least 1 L clear fluid, which was better tolerated and had superior mucosal cleansing than large-volume PEG + E (4 L PEG) alone [4]. The notable feature of our pretreatment was that we combined the preparations with a stimulant laxative, sennosides A and B, and an agent to improve bowel function, mosapride citrate. These methods enabled us to decrease the total liquid volume of both PEG + ascorbic acid and PEG + E preparations that had to be administered to patients, thus reducing the patients' burdens. In fact, our study showed that several tablets of bowel-stimulant laxative and digestive tract functional improvement agents, in combination with AJG522 preparations (not more than 2 L preparation volume), had a superior bowel cleansing efficacy and a greater ability to shorten the amount of bowel cleansing time. The shortening of the duration of bowel cleansing by the AJG522 preparation was able to decrease the length of the hospital visit for outpatients if the pretreatment for the colonoscopy was scheduled in a hospital, trimming the working hours of the medical staff preparing the colonoscopy, and finally contributing to higher labor productivity.

In view of the patients' feedback, only the acceptability was significantly different between the two groups. A possible explanation for this finding is that alternatively consuming the preparations and water was more acceptable than continuously ingesting the same preparation because the preparations are hard to drink in general. In this regard, we think that a significant decrease in the amount of preparation alone in the AJG522 group compared with the PEG + E group is very meaningful even though the total volume of preparations was not significantly different between the groups. As both preparations were made to suit the tastes of a Japanese population, it may be comprehensible that there was no significant difference regarding the taste preferences between the two groups. More patients answered “Satisfactory” in the AJG522 group than in the PEG + E group, although there was no significant difference between the groups regarding patient satisfaction as a whole. A possible reason for this is that the patient satisfaction demands were higher than expected. Possible reasons for the gap in the difference between “Acceptability” and “Willingness to repeat” include differences in the quality of the questions and a lack of choice regarding their preparation due to the assignment protocol. In other words, patients may tolerate the corresponding preparation but are unlikely to consume extra preparations, or patients may be likely to choose their next preparation of their own accord, regardless of the ease of consumption.

AJG522 has one disadvantage. It is a hyperosmolar solution that can cause dehydration by liquid moving to the intestine from the body. In fact, hemoconcentration was observed after taking AJG522 even though an amount of water equal to half of the volume of the AJG522 preparation was taken [7]. Therefore, AJG522 should be used with discretion for patients who need to be under close supervision regarding the amount of water ingested such as dialysis patients and heart failure patients.

The primary limitations of our study are related to its single-center nature and relatively small number of subjects, as this is only a pilot study. As mentioned above, the bowel preparation conditions in the colon lesions were not strictly evaluated.

## 5. Conclusions

The AJG522 preparation may be more efficient in bowel cleansing, more acceptable to patients, and able to significantly reduce the volume of preparation required. The clinical benefits of this practice seem to be independent of the nonreduced total volume of preparation the patients consumed in the AJG522 group compared to the PEG + E group. Large, multicenter prospective studies should be undertaken to confirm these results in the future.

## Figures and Tables

**Table 1:** Patient demographics, reasons for colonoscopy, and comorbidities.

Parameter	AJG522^*^	PEG + E^†^	Total
Number (%) of cases	32	33	
Age (yr)			0.369
Median	60	67	
Range	26–82	25–84	
Gender, *N* (%)			0.622
Male	16 (50.0%)	16 (48.5%)	
Reason for colonoscopy, *N* (%)			0.055
Abdominal pain	2 (6.3%)	4 (12.1%)	
Bloody stool	5 (15.6%)	6 (18.2%)	
Follow-up after polypectomy	5 (15.6%)	8 (24.2%)	
Diarrhea	0 (0%)	3 (9.1%)	
Constipation	1 (3.1%)	2 (6.1%)	
Tumor screening	3 (9.4%)	4 (12.1%)	
Anemia	2 (6.3%)	0 (0%)	
Inflammatory bowel disease	12 (37.5%)	4 (12.1%)	
Other	2 (6.3%)	2 (6.1%)	
Comorbidities, *N* (%)			0.518
Diabetes mellitus	5 (15.6%)	2 (6.1%)	
Hypertension	12 (37.5%)	5 (15.2%)	
Rheumatoid arthritis	2 (6.3%)	0 (0%)	
Chronic kidney disease	1 (3.1%)	0 (0%)	

^*^AJG522: the solution contained 100.0 g macrogol 4000, 7.5 g sodium sulfate, 2.7 g sodium chloride, 1.0 g potassium chloride, 4.7 g ascorbic acid, 5.9 g sodium ascorbate, and green apple flavoring per liter; total dose = 2 L. ^†^PEG + E (Niflec): the solution contained 59.0 g macrogol 4000, 5.7 g sodium sulfate, 1.5 g sodium chloride, 0.7 g potassium chloride, 1.7 g sodium bicarbonate, and lemon flavoring per liter; total dose = 2 L.

**Table 2:** Bowel cleansing efficacy.

	AJG522 (*N* = 32)	PEG + E (*N* = 33)	*P*
Bowel condition			
2 hrs^*^			0.013
Grade A	23 (71.9%)	14 (42.4%)	
Grade B	6 (18.8%)	8 (24.2%)	
Grade C	1 (3.1%)	9 (27.3%)	
Grade D	1 (3.1%)	0 (0%)	
No defecation	1 (3.1%)	2 (6.1%)	
3 hrs^†^			0.062
Grade A	28 (87.5%)	23 (69.7%)	
Grade B	4 (12.5%)	6 (18.2%)	
Grade C	0 (0%)	4 (12.1%)	
Grade D	0 (0%)	0 (0%)	
No defecation	0 (0%)	0 (0%)	
Liquid volume being taken (mL)^‡^			
Preparations	1000 (1000–2000)	2000 (1150–2000)	<0.0001
Water	500 (250–1000)	0	
Total	1500 (1250–3000)	2000 (1150–2000)	0.088

The bowel cleansing conditions at 2^*^ and 3 hours^†^ after taking the preparation.

^‡^Liquid volume being taken until the fecal properties reached grade A or B or for 3 hours after taking the preparations was shown in medians and ranges.

**Table 3:** Patients' feedback.

	AJG522 (*N* = 32)	PEG + E (*N* = 33)	*P*
Taste, *N* (%)			0.229
Good	9 (28.1%)	3 (9.1%)	
Satisfactory	8 (25.0%)	11 (33.3%)	
Neutral	7 (21.9%)	10 (30.3%)	
Unsatisfactory	7 (21.9%)	7 (21.2%)	
Bad	1 (3.1%)	2 (6.1%)	
Acceptability^*^, *N* (%)			0.010
Yes	21 (65.6%)	11 (33.3%)	
No	11 (34.4%)	22 (66.7%)	
Willingness to repeat, *N* (%)			0.347
Yes	11 (34.4%)	7 (21.2%)	
Acceptable	11 (34.4%)	12 (36.4%)	
Neutral	6 (18.8%)	12 (36.4%)	
No	4 (12.5%)	2 (6.1%)	
Patient satisfaction^†^, *N* (%)			0.145
Satisfactory	15 (46.9%)	8 (24.2%)	
Neutral	9 (28.1%)	15 (45.5%)	
Unsatisfactory	8 (25.0%)	10 (30.3%)	

^*^Acceptability was evaluated by the willingness to drink more preparation after the bowel cleansing conditions reached grade A or B or at 3 hours after taking the preparation if not. ^†^Patient satisfaction was evaluated by the satisfaction with the bowel preparation regarding time.
